# Murray Valley Encephalitis Virus: An Ongoing Cause of Encephalitis in Australia’s North

**DOI:** 10.3390/tropicalmed3020049

**Published:** 2018-05-11

**Authors:** John Floridis, Sarah L. McGuinness, Nina Kurucz, Jim N. Burrow, Rob Baird, Josh R. Francis

**Affiliations:** 1 Royal Darwin Hospital, Northern Territory, Darwin 0810, Australia; sarah.mcguinness@monash.edu (S.L.M.); jim.burrow@nt.gov.au (J.N.B.); rob.baird@nt.gov.au (R.B.); josh.francis@nt.gov.au (J.R.F.); 2 Centre for Disease Control, Northern Territory Department of Health, Northern Territory, Darwin 0810, Australia; nina.kurucz@nt.gov.au; 3 School of Public Health and Preventive Medicine, Monash University, Victoria, Clayton 3168, Australia; 4 Menzies School of Health Research, Charles Darwin University, Northern Territory, Casuarina 0811, Australia

**Keywords:** Murray Valley encephalitis virus, vector-borne, surveillance

## Abstract

Murray Valley encephalitis virus (MVEV) is a mosquito-borne virus endemic to Australia and New Guinea. Encephalitis due to MVEV is potentially devastating, and no therapeutic interventions of proven value exist. Prevention relies largely on personal protective measures against mosquito bites. We present a case of MVEV encephalitis with a favourable outcome following intensive care management and prolonged rehabilitation, and the epidemiological features of a further 21 cases notified to the health department of Australia’s Northern Territory. As cases occur in travellers, and epidemics occur sporadically in south-eastern Australia, clinicians across Australia and further abroad should be familiar with the disease and its diagnosis and management.

## 1. Introduction

Murray Valley encephalitis virus (MVEV) is a mosquito-borne flavivirus endemic to Australia and New Guinea. The virus is maintained in enzootic foci in the Top End, Katherine, and Barkly regions of the Northern Territory (NT) and northern Western Australia (WA) in a cycle involving waterbirds and *Culex annulirostris* mosquitoes [1,2,3]. Although *C. annulirostris* is considered the principal vector, *Aedes normanensis* is also believed to play a role in MVEV transmission [4].

Cases follow a seasonal pattern, with most cases occurring between February and June. The risk of human infection is highest in years with heavy wet season rains and extensive flooding [2,3]. Whilst the majority of infections are asymptomatic, between 1 in 150 and 1 in 1000 infections result in symptomatic disease [5]. Recognised clinical patterns of illness include relentless progression to death; prominent spinal cord involvement causing flaccid paralysis; brainstem involvement with cranial nerve palsy and tremor; and encephalitis followed by complete recovery [6]. Symptomatic disease is associated with a case fatality rate of 15–30%; 30–50% of survivors suffer long-term neurological sequelae [5]. No therapeutic interventions of proven value exist, and treatment is supportive. Prevention of disease relies on vector control measures, personal protective measures against mosquito bites, and flavivirus surveillance to detect virus activity [6].

MVEV is a nationally notifiable disease in Australia. In the NT, cases of MVEV are notified to the Centre for Disease Control in the NT’s Department of Health (DoH). Sentinel chicken flocks are used for flavivirus surveillance, acting as an early warning system to detect MVEV activity in the NT, and are maintained by the DoH’s Medical Entomology unit with regular virus testing carried out by the Department of Primary Industry and Resources. In the Alice Springs region, where MVEV is not believed to be endemic, a correlation has been shown between seroconversions in sentinel chickens, high summer rainfall >100 mm, mosquito vector numbers in overnight adult mosquito surveillance traps ≥300, and MVE disease cases [3]. We present a case of MVEV meningoencephalitis along with the epidemiological features of a further 21 cases notified to the NT DoH since the year 2000.

## 2. Case Report

An 8-year-old previously well Indigenous child from a remote NT community in the Katherine region presented with fever, seizures, and altered consciousness. There was no recent history of travel, insect bites, or animal exposures. On initial examination, he had a reduced Glasgow Coma Score (GCS) of 11 (Eyes 4, Verbal 2, Motor 5). Physical examination was otherwise unremarkable. He required intubation and intensive care unit admission due to persistent seizure activity and aspiration risk.

**Figure 1:**
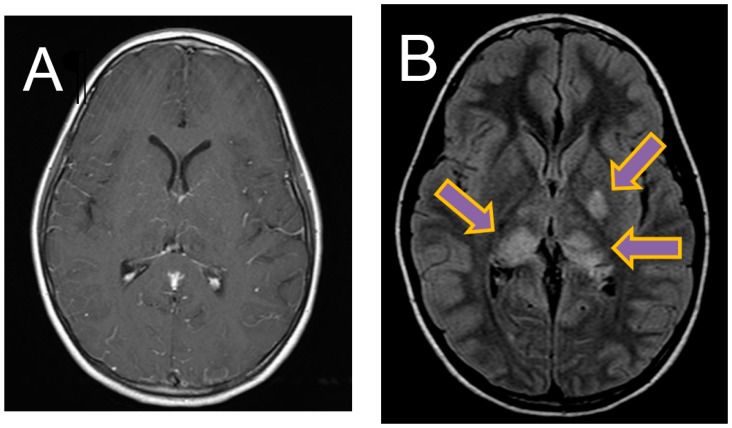
Magnetic resonance imaging (MRI) brain images. (**A**) Axial T1-weighted image showing diffuse leptomeningeal enhancement. (**B**) Axial FLAIR image showing FLAIR hyperintensity in left basal ganglia and bilateral thalami.

A lumbar puncture was performed and revealed a cerebrospinal fluid (CSF) leucocytosis (388 × 10^6^/L) with 80% polymorphonuclear cells, an elevated CSF protein of 0.86 g/L (NR 0.15–0.45 g/L), and CSF glucose of 2.7 mmol/L (NR 2.7–4.2 mmol/L). Magnetic resonance imaging (MRI) of the brain revealed diffuse leptomeningeal enhancement with T2 and FLAIR hyperintensity and restricted diffusion involving the left basal ganglia and bilateral thalami (Figure 1).

A broad differential diagnosis was considered and investigations were performed for common and regionally important aetiologies. A diagnosis of MVEV was confirmed by serologic and molecular methods: MVEV-specific polymerase chain reaction (PCR) was positive on CSF (confirmed by sequencing) and anti-MVEV IgM was detected in CSF and serum. Negative microscopy and culture of CSF excluded a concurrent bacterial infection, and empirical broad-spectrum antibiotics and antivirals were ceased. Neurological deficits became apparent following extubation, which included spastic paraplegia, dystonia, and deficits in self-care, language, and cognitive domains.

The patient had a prolonged admission, which was focused on supportive care, including controlled nasogastric feeding, pressure sore prevention, and early mobility exercises, with extensive input from allied health therapists. Following his 5-week acute pediatric admission, he was transferred to a dedicated multi-disciplinary pediatric rehabilitation service for 7 weeks of inpatient rehabilitation. The goals of his rehabilitation were to optimise his activities of daily living, social supports, mobility, nutrition, self-feeding, cognition, communication, behavior, and emotional wellbeing. Progress was evaluated both qualitatively and quantitatively using the WeeFIM^®^ score (Appendix B). His baseline WeeFIM^®^ score was 18.

His rehabilitation was complicated by the development of a right ankle contracture and intermittent impulsive behaviors. However, he proceeded to have a near-complete recovery of his deficits (WeeFIM^®^ score of 93 post-rehabilitation), and was able to return to his community.

## 3. Local Epidemiology and Surveillance

Twenty-two cases of encephalitis due to MVEV were notified to the NT DoH between 2000 and 2015 (Table A1). Onset of symptoms occurred between the months of February and July for all cases. Eighteen cases (82%) were exposed within the NT; 3 cases were exposed in WA, and one in South Australia. Amongst the identified cases, 12 were male (59%), 12 were Indigenous (59%), and 9 were children (41%). Six deaths (27%) resulted from the disease; two were children. All 9 children were Indigenous residents of an endemic area, whereas only 3 of the adults (23%) were Indigenous and 7 (54%) were local residents. Six cases occurred in adult travellers, including two international travellers. Both international travellers had devastating outcomes: one died [7] and the other developed permanent neurological sequelae, remaining partly ventilator-dependent with flaccid quadriparesis [8].

Virus testing of NT sentinel chicken flocks was carried out by the Department of Primary Industry and Resources, with MVEV seroconversions recorded every wet season except for 2012/2013, with seroconversions correlating temporally and geographically with 12 of the 18 cases exposed in the NT [9]. An excess of cases occurred in the years 2000 (7 cases) and 2011 (5 cases). Exceptionally high rainfall was seen in these years [10]. Sentinel chicken surveillance for MVE (and other flaviviruses) in 2000 showed activity throughout the Northern Territory (Alice Springs, Tennant Creek, Katherine, Darwin, and Gove) beginning in February, reaching a maximum in May, and abating by July [11].

## 4. Discussion

Encephalitis due to MVEV is potentially fatal and frequently causes long-term neurological sequelae; however, complete or near-complete recovery is possible as described in this case. In the NT, Indigenous children living in regional and remote areas appear to be most at risk. Few cases are reported in Indigenous adults, presumably due to high rates of MVEV exposure and subsequent seroconversion in childhood, as suggested by previous serologic studies [6,12]. Overseas and interstate travellers from non-endemic areas are at risk when travelling in the NT.

In enzootic areas, the highest risk period for MVEV infection is February to July. NT residents and tourists from non-endemic areas travelling to the NT in high-risk months are made aware of the risk of infection and preventative measures via media statements issued by NT DoH on an annual basis. Media statements are based on rainfall data, vector numbers, and MVEV seroconversions in sentinel chickens. Following the identification of two MVEV cases in Indigenous NT children in 2015, the NT DoH launched a radio and social media community awareness campaign in February 2016, with messages translated into and broadcast in all major Top End and Central Australian Indigenous languages, to increase awareness and reduce the potential for MVE cases in remote NT communities.

Vector monitoring and control measures are routinely performed in Darwin and other major NT centres, but these are not feasible on a state-wide scale. The use of sentinel chicken flocks in association with rainfall and vector numbers provide an adequate warning of virus activity and disease risk, with most seroconversions correlating temporally and geographically with cases. However, given the limitations in vector control, and the lack of an effective vaccination against MVEV, personal protective measures against mosquito bites, as advocated by Australian Health Departments, are vital to prevent infection.

## 5. Conclusions

MVEV remains an ongoing public health concern for residents of and travellers to northern Australia. This series of 22 cases of clinical MVEV infection highlights the potential severity of MVEV infection and reinforces the importance of providing education about personal protective measures to prevent mosquito bites to residents of, and travellers visiting, the NT and other endemic areas. The paediatric case presented illustrates the importance of coordinated supportive therapy and early rehabilitation for neurological recovery.

## Appendix A

**Table A1:** Demographics of clinical Murray Valley encephalitis virus (MVEV) cases notified to the local Centre for Disease Control Northern Territory.

Year	Onset Month	State of Exposure	Adult or Child	Indigenous Status	Sex	Residential Status	Diagnostic Method	Clinical Presentation & Outcome (Where Information Available)	Died
2000	March	NT	A	I	M	Resident	Serology (CSF)		No
2000	March	NT	C	I	F	Resident	Serology		No
2000	April	NT	C	I	M	Resident	Serology		No
2000	April	SA	A	I	M	Resident	Serology		No
2000	April	WA	A	N	M	International traveller	Serology	Flaccid quadraparesis, long-term impairment [8]	No
2000	May	NT	A	I	F	Resident	Serology		Yes
2000	May	NT	A	N	M	Traveller	Serology		No
2001	February	NT	A	N	F	Resident	Serology		No
2001	February	NT	A	N	M	Resident	Serology		No
2001	July	NT	C	I	F	Resident	Serology		No
2004	March	NT	C	I	F	Resident	Serology		Yes
2005	March	NT	C	I	M	Resident	Serology		No
2008	April	WA	A	N	M	Resident	Serology		No
2009	March	NT	A	N	M	Resident	Nucleic acid detection		Yes
2009	May	NT	A	N	M	Interstate traveller	Serology		Yes
2011	March	NT	C	I	M	Resident	Serology	Encephalitis, moderate residual neurological deficits [13]	Yes
2011	March	NT	A	N	M	Resident	Serology	Encephalitis, global neurological deficit [13]	No
2011	May	WA	C	I	F	Resident	Serology	Encephalitis, full recovery [13]	No
2011	May	NT	A	N	F	International traveller	Nucleic acid detection	Encephalitis, rapid progression to death [7,13]	Yes
2011	May	NT	A	N	F	Interstate traveller	Serology	Encephalitis, residual confusion [13]	No
2015	February	NT	C	I	F	Resident	Serology		No
2015	May	NT	C	I	M	Resident	Nucleic acid detection (CSF)	Encephalitis, near-complete recovery *	No

C = child, A = adult, I = Indigenous, N = non-Indigenous, M = male, F = female. NT = Northern Territory, WA = Western Australia, SA = South Australia. * Case described in this report.

## Appendix B

The WeeFIM^®^ is an outcome measure designed to provide an overview of a child’s or adolescent’s level of independence with everyday functional tasks. It is a measure of disability, and is intended to measure what the child actually does no matter what the diagnosis or impairment.

The WeeFIM^®^ incorporates 18 items that measure a child’s function and provides a basic indicator of severity of disability prior to and following participation in a rehabilitation program.

A seven-level scale is used to rate each item, with level seven indicating complete independence, through to level one, which indicates that the child requires total assistance from a helper or device to perform the task safely.
